# Aspirin and cancer treatment: systematic reviews and meta-analyses of evidence: for and against

**DOI:** 10.1038/s41416-023-02506-5

**Published:** 2023-11-29

**Authors:** Peter Elwood, Gareth Morgan, John Watkins, Majd Protty, Malcolm Mason, Richard Adams, Sunil Dolwani, Janet Pickering, Christine Delon, Marcus Longley

**Affiliations:** 1 Population Medicine, Cardiff University, Cardiff, CF14 4XN UK; 2 Systems Immunity Research Institute, Cardiff University, Cardiff, CF14 4XN UK; 3 School of Medicine, Cardiff University, Cardiff, CF14 4XN UK; 4 Wales Cancer Bank, University Hospital of Wales, Cardiff, CF14 4XN UK; 5 Freelance Statistician, London, UK; 6 University of South Wales, Cardiff, CF37 4BD UK

**Keywords:** Drug development, Combination drug therapy

## Abstract

Aspirin as a possible treatment of cancer has been of increasing interest for over 50 years, but the balance of the risks and benefits remains a point of contention. We summarise the valid published evidence ‘for’ and ‘against’ the use of aspirin as a cancer treatment and we present what we believe are relevant ethical implications. Reasons for aspirin include the benefits of aspirin taken by patients with cancer upon relevant biological cancer mechanisms. These explain the observed reductions in metastatic cancer and vascular complications in cancer patients. Meta-analyses of 118 observational studies of mortality in cancer patients give evidence consistent with reductions of about 20% in mortality associated with aspirin use. Reasons against aspirin use include increased risk of a gastrointestinal bleed though there appears to be no valid evidence that aspirin is responsible for fatal gastrointestinal bleeding. Few trials have been reported and there are inconsistencies in the results. In conclusion, given the relative safety and the favourable effects of aspirin, its use in cancer seems justified, and ethical implications of this imply that cancer patients should be informed of the present evidence and encouraged to raise the topic with their healthcare team.

## Introduction

In 1965, Sir Austin Bradford Hill set nine criteria against which a causal relationship between a presumed cause and an observed effect could be assessed [1]. These criteria are still useful, but Hill’s comment on his eighth criterion: ‘Experiment’: ‘*Occasionally it is possible to appeal to experimental evidence’* is somewhat dated. Now, thanks to Cochrane and others [2] the randomised controlled trial (RCT) is widely accepted as a ‘gold-standard’ within the hierarchy of evidence, and discussions about clinical interventions tend now to be dominated by whatever RCT evidence of clinical benefit is available. Randomised trials however have their own limitations and cannot give absolute certainty, so they therefore need to be considered in balance with other sources of evidence, including observational studies.

Hill’s criteria for a causal relationship also includes ‘Plausibility’ and in relation to aspirin and cancer, plausibility has been extensively established by the identification of effects of aspirin upon platelets and upon the many biological mechanisms relevant to cancer initiation, cancer metabolism, metastatic cancer spread, and thromboembolic complications in cancer [2].

In this review, we put together the published evidence from a wide range of sources which is favourable to the use of aspirin in cancer, and evidence that is unfavourable to its use. Finally, we urge the rights of patients with cancer to be sufficiently well informed about the risks and benefits of aspirin to enable them to raise the topic with members of their healthcare team, and to enable them, within discussion with their healthcare advisors, to decide whether or not to take the drug.

### For aspirin use in cancer

#### Aspirin, biological mechanisms and clinical outcomes

The primary mechanism of aspirin is inhibition of the cyclooxygenase (COX) enzyme responsible for the formation of key signalling lipids known as prostanoids. While this is an important pathway in cancer signalling, recent evidence highlights additional targets for aspirin in tackling cancer progression directly, irrespective of COX activity [3, 4]. Such targets include energy metabolism involved in cancer proliferation, cancer associated inflammation [5] and platelet driven pro-carcinogenic activity [2].

Aspirin was also shown to affect DNA repair pathways, which is a mechanism of particular interest in colorectal cancers. Defects in DNA mismatch repair genes are responsible for hereditary non-polyposis colorectal cancer (HNPCC), also known as Lynch syndrome [6], as well as other types of colorectal cancer by causing the occurrence of instability of simple repeat sequences (termed ‘microsatellite instability’) [7, 8] Colon cancers which exhibit a high microsatellite instability are currently targeted by immunotherapy treatment to a good effect [8].

However, aspirin has also been shown to play a role in reducing the occurrence of these microsatellite instabilities in cancer cell lines independent of COX activity, supporting a direct role for aspirin in DNA repair pathways in cancer [7]. These findings were further supported by a recent study by Nonu et al. [9] which combined proteomics and Mendelian randomisation to demonstrate a beneficial effect of aspirin on colon cancer risk through an enhancement of DNA-repair mechanisms [2].

Beyond genetic repair, aspirin has also been shown to influence epigenetic mechanisms relating to inflammation-associated cancer progression. In many cancers, inflammation leads to the promotion of carcinogenesis via direct mutagenesis or activation of a cytokine response, leading to the formation of ‘tumour microenvironments’ which are characterised by the presence of immune cells, stromal cells and extracellular matrix which together serve to promote cancer progression [10, 11].

One way in which inflammation leads to this cancer promotion is through epigenetic changes, the heritable transcription alterations that do not include changes in DNA sequence [12]. Aspirin has been shown to interfere with these epigenetic cancer-related changes by mediating histone methylation, leading to an in vivo decrease of tumour growth and metastasis in animal models of metastatic cancer through interfering with cells in the tumour microenvironment [12, 13].

Together, these observations provide mechanistic evidence for the causal involvement of aspirin in modifying cancer pathways, DNA repair mechanisms as well as epigenetic mechanisms which jointly provide a ‘basic science’ basis to justify using aspirin as an adjunct to other pre-existing therapies (e.g., immunotherapy and cytotoxic chemotherapy) in the treatment of cancer progression and metastasis [2, 14].

#### Aspirin and vascular complications in cancer

Aspirin has been shown repeatedly to reduce thromboembolism, including in patients with cancer [15], and the biological mechanisms through which this is achieved have been well described [2].

In the UK records for 108,000 survivors of cancer were examined. Venous thromboembolism and other vascular causes of death were found to be substantially elevated in patients with almost all the cancers and were most pronounced in patients who had received chemotherapy [16].

In the USA the SEER programme on mortality in cancer patients reported that 11% of deaths amongst patients with twenty different cancers had been certified as due to vascular disease, most of which (76%) was heart disease [17]. This led the American Society of Clinical Oncology to recommend that prophylactic anticoagulants be considered for all hospitalised cancer patients [18]. Although aspirin use has to some extent been superseded by recently developed drugs for vascular protection, aspirin is still effective against thrombosis, including venous thrombosis [19].

#### Aspirin and metastatic cancer spread

The effect of aspirin on metastatic spread is of importance because metastases are responsible for much of the pain and the complications of cancer [20] and many of the deaths are attributable to metastases [21]. Platelets play a significant role in metastatic spread, and the relationship between these effects and the clinical outcomes has been detailed elsewhere [2, 22].

There appears to have been no systematic literature search and meta-analysis of clinical data on metastatic cancer, but many studies and overviews give evidence of substantial reductions by aspirin (ranging from about RR 0.48 (95% CI 0.30 to about 0.75), to RR 0.62 (0.52 and 0.75) [23, 24].

It is important to note that an effect on cancer spread indicates a value of aspirin that is independent of its effects upon cancer mortality [24–26]. Indeed any delay in the diagnosis and initiation of treatment would seem to make a reduction in metastatic spread of increased value.

#### Aspirin and cancer mortality

An effect of aspirin of particular interest is its enhancement of the mismatch repair of DNA [3], a protective mechanism against cancer within all of us. Failure of this mechanism leads to Lynch Syndrome, with a high risk of colon and other cancers and with an estimated prevalence of one in 279, or 0.35% in the general population [27].

Following the report of a reduction in mortality by aspirin in patients with this syndrome [28], the National Institute of Clinical Excellence in the UK judged the safety and effectiveness of aspirin favourable and recommends aspirin for the reduction of cancer in patients with the Syndrome [29].

A systematic literature search in 2016, together with two replicate searches in 2018 and in 2021 identified 118 published observational studies of cancer patients, representing about 1 million patients with 18 different cancers [30]. About a quarter of these patients reported taking aspirin at diagnosis (most usually for vascular protection, and therefore 75 or 81 mg daily) and a pooled analysis showed a reduction of 21% in all-cause mortality (HR 0.79; 95% CI 0.74, 0.86 in 56 reports that used hazard ratios and OR 0.57 (0.36, 0.89) in seven papers that reported odds ratios). Table 1 provides a summary of the key data.

**Table 1:** Aspirin taking and mortality: meta-analysis of 118 published observational reports [30].

Group	Cancer mortality		All-cause mortality	
	No. of studies	HRs (95% CIs)	No. of studies	HRs (95% CIs)
		ORs (95% CIs)		ORs (95% CIs)
Colon cancer	24	HR 0.71 (0.62, 0.80)	21	HR 0.81 (0.73, 0.91)
	1	OR 0.78 (0.66, 0.93)	1	OR 0.78 (0.65, 0.92)
Breast cancer	13	HR 0.84 (0.72, 0.98)	9	HR 0.94 (0.70, 1.25)
	4	OR 0.75 (0.36, 1.57)	None	–
Prostate cancer	15	HR 0.89 (0.78, 1.02)	6	HR 1.00 (0.78, 1.27)
	1	OR 1.02 (0.78, 1.34)	1	OR 1.06 (0.94, 1.19)
15 other cancers	18	HR 0.79 (0.70, 0.83)	21	HR 0.67 (0.60, 0.75)
	5	OR 0.49 (0.26, 0.95)	5	OR 0.47 (0.26, 0.83)
All 18 cancers	70	HR 0.77 (0.72, 0.83)	56	HR 0.79 (0.74, 0.86)
	11	OR 0.67 (0.45, 1.00)	7	OR 0.57 (0.36, 0.89)

Publication bias, arising from the selective publication of positive findings for an intervention such as aspirin, is a most important issue in meta-analyses such as the above. A ‘trim and fill’ testing procedure to restore symmetry in forest plots was therefore applied extensively to the data, and although the benefits of aspirin were reduced, significance of almost all the reductions remained [30].

In their report the authors identified 23 publications which had focused upon fifteen less studied cancers (naso- and oropharyngeal, oesophagus, gastrointestinal, gastric, rectal, liver, gallbladder, bladder, pancreas, bladder, endometrium, ovary, glioma, head and neck, lung, melanoma). Meta-analysis of these gave pooled reductions of around 30% in deaths associated with aspirin taking (HR 0.67; 0.60, 0.75 in 21 studies, and OR 0.47; 0.26, 0.83 in five studies) [30]. Together with the evidence of favourable effects of aspirin upon a wide range of biological mechanisms relevant to cancer mortality [3], these clinical outcomes suggest that aspirin is likely to be of benefit to patients within a very wide range of different cancers.

Unfortunately, in contrast to observational studies, very few randomised trials of aspirin and mortality have been reported. The pooled results of four early trials based upon a total of 722 patients with cancer gave a suggestive pooled reduction associated with aspirin of about 9% in cancer deaths [30]. Currently, a number of randomised trials which test aspirin and mortality are in progress. These focus upon the common cancers: colon, breast, prostate and one in lung cancer. One of these trials, based upon 3021 selected patients in remission from a HER2-negative breast cancer, has already reported [31]. This trial was ended prematurely because aspirin was associated with a possible increase of about 25% in deaths.

The opportunity to conduct long-term follow-up studies of deaths in subjects who had already participated in randomised trials of aspirin and vascular disease was taken by Rothwell and colleagues in Oxford. Subjects who had been involved in up to 51 randomised vascular trials were followed-up for up to 20 years. Consistently, a reduction in cancer deaths was shown in these studies: (OR 0.58; 95% CI 0.44, 0.78 in an overview of six vascular trials [23], and OR 0.84; 0.75, 0.94) in an overview of 51 randomised vascular trials) [32].

An opportunistic trial was conducted in a subset of subjects who were participants in a randomised trial of prophylactic aspirin. During the study 502 subjects developed cancer of the prostate, and follow-up of these showed a 30% relative reduction attributable to aspirin (HR 0.68, 95% CI 0.52, 0.90 in cancer deaths and HR 0.72; 0.61, 0.9 in all-cause deaths) [33], and the evidence given by aspirin taken by patients with the Lynch syndrome [27], together with Mendelian randomisation studies, powerfully supplement the evidence available from conventional randomised trials.

#### Aspirin and the duration of survival

A few authors report estimates of the length of additional survival associated with aspirin taking by patients with cancer. A number of different summary statistics of survival have been used, and these defy pooling, but the additional survivals range from about 3 months up to 3 years [30].

Using a different approach, a group in Liverpool extracted extensive baseline data, including aspirin taking, from the records for 44,000 patients with colon cancer. With these they constructed a formula giving predicted estimates of survival [34]. Entering into the formula the details for a typical non-diabetic patient aged 70 with colon cancer, the inclusion of aspirin increases the estimate of survival by about 5 years for a man, and about 4 years for a woman.

### Against aspirin use in cancer

#### Additional gastrointestinal bleeding

A bleed, either gastrointestinal or intra-cerebral, is a crisis for a patient and especially for patients who are already seriously ill [35–37]. Yet the seriousness of bleeds attributable to aspirin, and not just their frequency, should be evaluated against the benefits attributable to the use of aspirin [38]. The most serious bleeds are those that are responsible for death, and survival/death is a clear dichotomy, requiring no value judgement.

In a systematic literature search eleven randomised trials which included data on fatal bleeding were identified [39]. These 11 RCTs included together a total of 121,094 subjects, followed for an average of 2.8 years, as shown in Table 2.

**Table 2:** GI bleeding in a meta-analysis of data from 11 RCTs [39] (average duration 2.8 years).

Bleeding	Risk of a bleed (in 2.8 years)	Relative risk for aspirin
Incidence of a GI bleed		
- in 54,625 subjects randomised to aspirin	8 per 1000	RR 1.55
- in 52,583 subjects randomised to placebo	5 per 1000	(1.32, 1.83)
Proportion of bleeds that were fatal		
- in subjects on aspirin	4%	RR = 0.45
- in subjects on placebo	8%	(0.25, 0.80)
Risk of a fatal bleed in trial participants		
- randomised to aspirin	3.7/10,000	RR = 0.77
- randomised to placebo	4.7/10,000	(0.41, 1.43)

These data confirm the usual excess risk of all ‘major’ bleeds for aspirin (RR 1.55), equivalent to about one per bleed per 1000 persons per year. Note however that the 50% increase above the background risk of bleeding from a peptic ulcer, stomach infection or other pathology, means that amongst patients who are taking aspirin the risk of a GI bleed being truly attributable to aspirin is only one in every three bleeds.

However, the proportion of ‘major’ bleeds in subjects who had been randomised to aspirin that led to death was 4% in those who had been randomised to aspirin while 8% of bleeds in subjects randomised to placebo were fatal. Clearly, this implies that overall, the bleeds truly attributable to aspirin must be of a much lower severity than other bleeds attributable to stomach pathology. This is further confirmed by the absence of any increased risk of a fatal bleed associated with aspirin taking, as shown in the third cell in the above table (RR 0.77), and this final conclusion has been confirmed in overviews of bleeding reported by other authors [39].

It is unfortunate however that the scientific literature on the issue of aspirin and bleeding appears to have been swamped by a host of statements about serious dangers of aspirin, most unsupported by any evidence while some are total misinterpretations. Probably the most misleading and most influential item on the web was a report issued by Reuters on the 14th June 2017, stating: ‘daily aspirin causes 3000 deaths from bleeding in Britain every year’ [40]. This claim was taken up and very widely and repeatedly publicised in the web and the media across the world. The report by Reuters was however a totally invalid, having been based on a prospective study of 3166 older patients, all of whom (93–97%) were taking aspirin. There were therefore no control subjects and no valid estimate of the independent contribution of aspirin to the fatal bleeds can be made.

In addition to this, there have been reports of so-called ‘neurogenic’ bleeding in patients with acute ischaemic strokes. A report from six thousand patients in the Fukuoka Stroke Registry describes 89 patients (1.4%) who experienced a GI bleed within a week of admission for acute ischaemic stroke [41]. O’Donnell et al. reported an incidence rate of 1.5% within a week of admission for acute ischaemic stroke, associated with a high rate of death [42] and Davenport et al. estimated an incidence of 3% [43]. In the study which led to the Reuters claim of 3000 deaths 2000 (65%) of the patients had had a stroke!

#### Additional cerebral bleeding

Unlike a gastrointestinal bleed, the consequences of a cerebral bleed, whether or not fatal, can be of a severity comparable to a cancer or a myocardial infarct in a risk/benefit evaluation. Estimates of additional risk in patients on aspirin are around one or two events per ten thousand (10,000) subject-years [35, 44, 45]. The major factor in cerebral bleeding however is hypertension [46], and in an RCT of aspirin based on more than 18,000 hypertensive patients—all of whom were receiving ‘optimal’ antihypertensive treatment—there were no additional cerebral bleeds in patients randomised to aspirin [47].

#### Inadequate support from randomised trials of aspirin

At present, the strength of the case for the use of aspirin in cancer lies in the wealth of evidence of benefit in observational cohort and case-control studies of aspirin taken by patients with cancer, while support from RCTs is seriously limited to a small number of trials, and there are serious inconsistencies between these.

There are calls by many for a delay on the promotion of aspirin until there is better and more consistent evidence from randomised trials. However, one seriously questions how much evidence, from how many randomised trials, in how many different cancers, will be required to resolve the uncertainties in the pooled observational studies.

## Discussion

The first ethical principle in clinical practice is: do no harm: non-maleficence. In the evaluation of excess bleeding attributable to aspirin, the absence of any valid evidence of fatal bleeding (see Table 2 and the related references) is reassuring and indicates that evaluated against cancer, or a thrombotic vascular event, aspirin is a reasonably safe drug. This conclusion is supported by the recommendation of aspirin by NICE as a treatment for some patients at risk of cancer [29].

Beneficence—perhaps the second most important ethical principle of relevance to clinical interventions—has been established with difference levels of probability for the three main clinical effects of aspirin. A reduction in thromboembolic events has been widely and repeatedly established with a high level of certainty, and a reduction in metastatic cancer spread seems to be a reasonable expectation based upon both clinical reports and the effect of aspirin upon relevant biological mechanisms. While the evidence for a reduction in mortality lacks consistent support from RCTs, both the evidence of benefit in Lynch syndrome [12], and further evidence from Mendelian randomisation studies [2] give considerable support.

Furthermore, the effect of aspirin on both the biological mechanisms relevant to thromboembolism and to metastatic cancer spread, are different to the biological mechanisms of aspirin and cancer growth and survival. This seems to indicate that aspirin is a useful cancer treatment whether or not the drug does truly affect survival.

However, the wealth of favourable evidence on aspirin and mortality in observational cohort and case-control studies of patients with cancer cannot be lightly dismissed. Granted, confounding by unknown factors independent from aspirin is possible and perhaps even likely, yet the evidence from Mendelian randomisations studies powerfully supplements the few results from randomised trials.

The situation with cancer in the poorer countries is clearly ethically unjust. One in every six deaths worldwide is due to cancer [48], giving an estimated 9.6 million deaths in 2018, with around 70% of the deaths in low- and middle income- countries [49]. WHO points out that most cancers in the poorer countries are diagnosed at a very late stage, when most treatments are no longer effective—even if treatments were available, which they are not in many countries [50]. Against that background the promotion of aspirin would be of enormous benefit in developing countries.

The ethical issue of autonomy concerns the right of a patient to be involved in every aspect of his or her care and treatment [51, 52]. Aspirin is inexpensive and readily available globally. It is easily taken with none of the highly distressing effects that accompany some of the cancer therapies. While aspirin should best be considered as a possible adjunct treatment for cancer, yet for those patients who refuse the more aggressive treatments, and for patients for whom palliative care is judged to be appropriate, aspirin should be considered.

Given the relative safety of aspirin; given its likely reduction in metastatic cancer spread; given its associated reduction in thromboembolic complications and given the support by NICE for aspirin use in a subset of cancers, is it ethically reasonable for patients to be kept in ignorance about the probable risk/benefit balance of aspirin?

Early in this work, in 2010, a challenge was published in the BMJ: “The debate about aspirin has consumed the medical profession for over 30 years, [*now, almost 50 years!*] yet almost no public participation or consultation has occurred” [53]. In response, a 3-day far ranging enquiry—a ‘Citizens’ Jury—under the general title: ‘My Health—*whose responsibility?’* was held in Cardiff with members of the general public who had no vested interest in the topic [54]. Over several days, the jury listened to a range of (sometimes contradictory) expert evidence, and the evidence of ‘experts by experience’, and vigorously debated amongst themselves the various issues raised. An immediate outcome of this initiative was a verdict by the sixteen members of the ‘jury’ that patients and the public should be directly involved in the evaluation of the outcomes of research, and in the assessment of its relevance to clinical practice and to public health policy…. and to this last the jurors unanimously added the phrase: ‘*even before there is agreement between doctors’* [54].

In the UK, the NHS Ethical Clinical Guidelines establish that people have a right to be involved in discussion and have a right to make informed decisions about their care [55]. However the law in the UK goes further and in a ‘Landmark Decision’ given by the UK supreme court in 2015 it was stated: ‘If information is material, doctors should generally disclose it. They should not wait for the patient to ask’ [56]. Surely evidence on the possible benefits of aspirin are highly ‘material’ to patients with cancer and to their carers!

*‘Medicine is a science of uncertainty and an art of probability.’*

Sir William Osler (1849-1919)

Frequently described as the father of Modern Medicine. (1849-1919)

## Conclusions

A major strength of the case for the promotion of aspirin as a treatment of cancer lies in the consistent evidence of a reduction in the thromboembolic complications of cancer and in the consistent evidence of a reduction in metastatic cancer spread. The main weakness, however, lies in the lack of support of a reduction in deaths from trials with random allocation of aspirin. However, the suggestive evidence from observational studies, together with evidence from Mendelian randomisation powerfully favour the use of low-dose aspirin.

Finally: aspirin is inexpensive, readily available and has none of the highly aggressive side effects of some of the cancer treatments. It would therefore seem to be only fair and reasonable that knowledge of the true risk and probable benefits of the drug should be widely publicised amongst cancer patients and their carers—so that, as one oncologist has predicted:

There could be benefit ‘…both to the affluent and the indigent within developed and under-developed countries’… and…’a truly global impact on cancer mortality could be realised [57].
